# Identification of environmental Actinobacteria in buildings by means of chemotaxonomy, 16S rRNA sequencing, and MALDI-TOF MS

**DOI:** 10.1128/spectrum.03596-23

**Published:** 2024-02-01

**Authors:** Anna Chudzik, Kaisa Jalkanen, Martin Täubel, Bogumiła Szponar, Mariola Paściak

**Affiliations:** 1 Hirszfeld Institute of Immunology and Experimental Therapy, Polish Academy of Sciences, Wroclaw, Poland; 2 Environmental Health Unit, Finnish Institute for Health and Welfare, Kuopio, Finland; Texas A&M University, College Station, Texas, USA

**Keywords:** MALDI-TOF MS, species identification, chemotaxonomy, 16S rRNA, Actinobacteria, *Streptomyces*

## Abstract

Actinobacteria are abundant in soil and other environmental ecosystems and are also an important part of the human microbiota. Hence, they can also be detected in indoor environments and on building materials, where actinobacterial proliferation on damp materials can indicate moisture damage. The aim of this study was to evaluate the matrix-assisted laser desorption ionization–time of flight mass spectrometry (MALDI-TOF MS) for the identification of 28 environmental strains of Actinobacteria isolated from building materials and indoor and outdoor air samples, mainly collected in the context of moisture damage investigations in buildings in Finland. The 16S rRNA gene sequencing and chemotaxonomic analyses were performed, and results were compared with the MALDI-TOF MS Biotyper identification. Using 16S rRNA gene sequencing, all isolates were identified on the species or genus level and were representatives of *Streptomyces, Nocardia,* and *Pseudonocardia* genera. Based on MALDI-TOF MS analysis, initially, 11 isolates were identified as *Streptomyces* spp. and 1 as *Nocardia carnea* with a high identification score. After an upgrade in the MALDI-TOF MS *in-house* database and re-evaluation of mass spectra, 13 additional isolates were identified as *Nocardia*, *Pseudonocardia,* and *Streptomyces*. MALDI-TOF MS has the potential in environmental strain identification; however, the standard database needs to be considerably enriched by environmental Actinobacteria representatives.

**IMPORTANCE:**

The manuscript addresses the challenges in identifying environmental bacteria using matrix-assisted laser desorption ionization–time of flight mass spectrometry (MALDI-TOF MS) Biotyper-based protein profiling. The matter of the studies—actinobacterial strains—has been isolated mostly from building materials that originated from a confirmed moisture-damaged situation. Polyphasic taxonomy, 16S RNA gene sequencing, and MALDI-TOF mass spectrometry were applied for identification purposes. In this experimental paper, a few important facts are highlighted. First, Actinobacteria are abundant in the natural as well as built environment, and their identification on the species and genus levels is difficult and time-consuming. Second, MALDI-TOF MS is an effective tool for identifying bacterial environmental strains, and in parallel, continuous enrichment of the proteomics mass spectral databases is necessary for proper identification. Third, the chemical approach aids in the taxonomical inquiry of Actinobacteria environmental strains.

## INTRODUCTION

It is estimated that people spend approximately 90% of their lives in built environments, making it necessary to control indoor airborne microorganisms as part of efforts to maintain good indoor air quality and health-promoting indoor environments. In addition to human (and animal) occupants, major sources of indoor bacteria are both the outdoor environment and potentially the buildings themselves (1). Actinobacteria, a phylum of Gram-positive bacteria with high G + C content, are abundant in soil and other environmental ecosystems and are part of the human microbiome as well. They have been reported in various indoor environments and different sample types: indoor air, surfaces, dust, and building materials (2), building materials (3), and air (4, 5). Strains of many Actinobacteria genera have been isolated from indoor air and building materials by culture-dependent methods, among others: *Brevibacterium, Corynebacterium, Kocuria, Micrococcus, Mycobacterium, Nocardia, Nocardiopsis, Pseudonocardia, Rhodococcus, Saccharopolyspora,* and *Streptomyces* (4). In moisture-damaged buildings, Actinobacteria of certain genera can grow together with fungi on wet building materials, and Actinobacteria overgrowth indoors can indicate moisture damage (6, 7). Excessive indoor microbial exposure due to moisture damage is considered a health hazard by the Finnish legislation (8), but the guidance is precautionary, and limit values are not health-based. An analysis of building material samples is performed if moisture-related microbial growth cannot be assessed by visible investigation but is suspected. Methods used to evaluate bacteria and fungi colonizing building materials are based on cultivation and define microbial concentration above the limit value or by an increased concentration of fungi combined with the manifestation of moisture indicator families or groups of fungi and/or Actinobacteria (9). The abundance of spore-forming Actinobacteria is confirmed by the detection of colonies of specific morphology and aerial hyphae observed in optical microscopy. The robust identification of Actinobacteria isolates on the genus and species level is not trivial and is time-consuming, while it could potentially aid building assessments, for example, if it would enable to more accurately differentiate moisture damage-associated Actinobacteria from outdoor environmentally sourced strains.

Moreover, the identification of Actinobacteria from indoor samples would allow the performance of targeted investigations into associations with occupants’ health. While associations between exposure to moisture damage and adverse, especially respiratory, health effects are well established (10–12), the mechanisms underlying these effects and the causative agents involved, including the role of specific fungal or (actino)bacterial taxa, are insufficiently understood.

The present study aimed to evaluate the MALDI-TOF MS system alongside chemotaxonomic analysis and 16S rRNA gene sequencing for the identification of environmental strains of Actinobacteria. The strains were isolated from building materials and originated from confirmed moisture-damaged situations, as well as from other indoor and outdoor air samples. The results of chemotaxonomic analyses (i.e., characteristics of polar lipids, fatty and mycolic acids, and amino acids of peptidoglycan) were compared with the data obtained by MALDI-TOF mass spectrometry and the Bruker Biotyper database and integrated with 16S rRNA gene sequencing.

## MATERIALS AND METHODS

### Sampling, cultivation, and isolation of bacterial strains

Bacterial strains were isolated from samples of building materials and indoor and outdoor air collected in Finland between July 2017 and June 2019 (Table 1). Twenty strains were isolated from 17 building material samples, originating from 15 different buildings. These samples were collected by individual customers or trained civil engineers in the context of building investigations to assess moisture damage and microbial growth and were sent to the Finnish Institute for Health and Welfare in Kuopio for cultivation analysis (Table 1). In addition, seven strains were collected from five indoor air samples taken from one terraced house and four detached houses as part of a research project on moisture damage. One strain (AKT34) originated from an outdoor air. Air samples were collected with an Andersen 6-stage impactor (13), with an air flow of 28.3 L/min and sampling time indoor and outdoor of 10 and 5 min, respectively.

**TABLE 1:** Source isolation of the strains studied

Strain no.	PCM no.	Sample	Type of material	Comment	Isolation year
AKT01	3204	Building material	Litter insulation		2017
AKT02	3205	Building material	Sowdust insulation (wood)		2017
AKT03	3206	Building material	Insulation material		2017
AKT04	3207	Building material	Insulation material		2017
AKT05	3208	Building material	Wood		2017
AKT06	3209	Building material	Concrete		2017
AKT07	3210	Building material	Mineral wool insulation		2017
AKT08	3211	Building material	Glass wool insulation		2017
AKT09	3212	Building material	Glass wool insulation		2017
AKT10	3213	Building material	Mineral wool insulation		2018
AKT11	3214	Building material	Mineral wool insulation		2018
AKT12	3215	Indoor air		Extracted from same building as AKT10	2018
AKT13	3216	Indoor air		Extracted from same building as AKT16	2018
AKT16	3217	Building material	Mineral wool insulation		2018
AKT17	3218	Building material	Mineral wool insulation		2018
AKT18	3219	Building material	Mineral wool insulation	Extracted from same sample as AKT17	2018
AKT19	3220	Building material	Mineral wool insulation		2018
AKT20	3221	Building material	Mineral wool insulation	Extracted from same sample as AKT19	2018
AKT21	3222	Building material	Mineral wool insulation	Extracted from same sample as AKT19	2018
AKT22	3223	Indoor air		Extracted from same building as strain AKT19	2018
AKT24	3224	Building material	Mineral wool insulation		2018
AKT25	3225	Indoor air			2018
AKT26	3226	Indoor air			2018
AKT27	3227	Indoor air		Extracted from same sample as AKT26	2018
AKT28	3228	Indoor air		Extracted from same sample as AKT26	2018
AKT34	3229	Outdoor air			2018
AKT39	3230	Building material	Mineral wool insulation		2018
AKT42	3231	Building material	Wallpaper and cement		2019

The viable bacteria and fungi were determined at the Finnish Institute for Health and Welfare according to the Finnish Decree on Housing Health (14) and National Supervisory Authority for Welfare and Health (9) recommendation. Briefly, samples of building materials were weighed (1–5 g); cut into pieces with sterile knives, scissors, or tweezers; and extracted with sterile dilution buffer (distilled water with 42.5 mg/L KH_2_PO_4_, 250 mg/L MgSO_4_ × 7H_2_O, 8 mg/L NaOH, and 0.02% Tween 80). Suspensions were sonicated (FinnSonic bath, MO3/m) for 30 min and shaken for 60 min (600 rpm/min) (Mini Shaker, VWR). Serial dilutions were made with the dilution buffer (as above), and 100 µL of aliquots was spread on two fungal media: 2% malt extract agar (MEA) and dichloran‐glycerol 18 agar (DG18) with chloramphenicol (0.1%) to restrain the bacterial growth. Total viable mesophilic bacteria and Actinobacteria were counted on tryptone yeast extract glucose agar (TYG) with natamycin (0.2%) to restrain the fungal growth. Air samples were collected with an Andersen 6-stage impactor on the same agar media.

Samples were incubated in the dark at 25°C for 7 and 14 days, respectively. Total counts of mesophilic and xerophilic fungi to the genus level were performed from MEA and DG18 media with an optical microscope. The total count of mesophilic bacteria was determined on TYG media, and actinomycetes-type bacterial colonies were separately counted with respect to their morphological features (typically white or grayish colonies, with a matte or powder surface) and microscopic observation of aerial hyphae. Pure actinobacterial colonies were cultured on TYG media, suspended in 20% glycerol, and stored at −80°C. The isolated actinobacterial strains were then deposited in the Polish Collection of Microorganisms (PCM) (Table 1).

### Reference bacterial strains

For comparative analyses in chemotaxonomic studies, the following collection strains from PCM were used: *Streptomyces griseus* PCM 2331 (DSM 40855), *Rhodococcus equi* PCM 559^T^ (DSM 20307^T^), *Nocardia farcinica* PCM 2712^T^ (DSM 43665^T^), *Nocardia abscessus* PCM 3042, and *Tsukamurella paurometabola* PCM 2453^T^ (ATCC 8368^T^).

### Chemotaxonomic methods

All but one isolate was cultivated on tryptic soy broth in the orbitally shaken flasks for 48 h at 25°C, to obtain bacterial biomass; the isolate AKT7 was cultivated for 5 days because of slow growth. Bacteria were inactivated in the Koch apparatus (1 h, 100°C), centrifuged at 6,000 rpm (Sigma), and washed twice with phosphate-buffered saline (PBS) and water. The wet biomass was freeze-dried.

The diaminopimelic acid (DAP) content in whole-cell hydrolysates was determined according to reference (15) and analyzed by thin-layer chromatography (TLC) with 2,6-diaminopimelic acid standard and N-glycolylated muramic acid by the colorimetric method (16). Fatty acid and polar lipid analyses were performed according to reference (17). Fatty acids were analyzed by gas chromatography/mass spectrometry (GC/MS) on Focus GC connected with Ion Trap ITQ 700, with Rxi-5 ms (30 m × 0.25 mm × 0.25 µm) column and Agilent GC 7890b spectrometer 700D, DB 5ms 30m (30 m × 0.25 mm × 0.25 µm) column, in triplicate.

Glycolipids were analyzed on TLC using a solvent system: chloroform–methanol–water (65:25:4, vol/vol/vol) and visualized using an orcinol reagent (18). Phospholipids were analyzed by one- and two-dimensional TLC with phospholipid standards. TLC plates were developed in two directions (I and II): the first in the system containing chloroform, methanol, and water (65:25:4, vol/vol/vol) and the second containing chloroform, acetic acid, methanol, and water (80:15:12:4, vol/vol/vol/vol). Dittmer and Lester’s reagent was used for development, which enabled the visualization of phospholipids. Mycolic acids were obtained by acid hydrolysis and the alkaline method according to reference (19) and analyzed on TLC with authentic mycolate standards.

### 16S rRNA gene sequencing

DNA was extracted from pure microbial cultures using a Chemagic Plant DNA kit with a preceding bead-beating step for mechanical cell disruption (20). DNA amplification of the 16S rRNA gene using primers 27F and 1492R, as well as Sanger sequencing, was done at commercial sequencing partner LGC Genomics (GmbH, Berlin, Germany). Amplification was performed using the MyTaq DNA Polymerase Kit (Bioline) and Biostab (PCR Optimizer; Bitop AG). PCR quality control was done via agarose gel electrophoresis, followed by ExoSAP-Purification. Sequencing was performed with BigDye Terminator v3.1 (Thermo Life Technologies) on a 3730xl DNA Analyzer. The sequences were blasted against the NCBI sequence database (16S ribosomal RNA) (Bacteria and Archaea type strains, accessed 14 July 2022) for the identification of database entries with highly similar 16S rRNA gene sequences. Sequence alignment including isolates and reference sequences and analysis of evolutionary relationships of taxa were performed in MEGA X (21). The evolutionary history was inferred using the neighbor-joining method, and the optimal tree (500 replicates in the bootstrap test) was calculated. The evolutionary distances were computed using the *p*-distance. All ambiguous positions were removed for each sequence pair (pairwise deletion option).

### MALDI-TOF MS

For MALDI-TOF MS analysis, actinobacterial isolates were cultivated on nutrient agar (NA), brain heart infusion agar (BHI), sheep blood agar (BL), tryptic soy–thioglycollate agar (TS), and yeast extract glucose agar (medium 79) (17) and were grown at 25°C for 2–7 days. The following sample preparation methods were used in the MALDI-TOF MS analysis. The direct colony transfer method (DT) was a simple collection of colonies from an agar plate using a sterile loop and applying it directly to a steel target MALDI plate (MTP 384 target plate). One microliter of HCCA matrix solution (alpha-cyano-4-hydroxycinnamic acid, HCCA, dissolved in 50% acetonitrile with 2.5% trifluoroacetic acid) was then applied to the dry sample. Direct colony transfer modified with formic acid treatment on the target plate (DTFA) was performed by adding 1 µL of 70% formic acid (FA) on top of the dry sample, followed by overlaying it with 1 µL of the matrix solution (22).

The ethanol–formic acid extraction (EFAE) procedure (recommended by the manufacturer) was also used: briefly, colonies from a solid medium were collected with a sterile loop and suspended in 300 µL Milli-Q water in an Eppendorf tube using a micropestle and shaken for 1 min (Vortex). Then, 900 µL of ethyl alcohol was added and vortexed again for a minute. The cells were centrifuged (1,300 rpm for 2 min); then, the supernatant was removed, and the remaining cells were left to dry. An extraction with 70% formic acid and acetonitrile was performed, and after centrifugation, 1 µL of an analyte was applied to the MALDI target, dried, and overlaid with 1 µL of the matrix solution.

MALDI-TOF MS analysis was conducted on the Ultraflex mass spectrometer (Bruker Daltonics, Germany) using Biotyper 3.1 software and a database containing 6,904 entries. Spectra were recorded in the linear positive ion mode within a mass range of 2,000–20,000 Da. The sum spectra of 2,800 laser shots were acquired in portions of 700 laser shots from four different spot positions. The identification criteria used in the analysis, formulated by the manufacturer, were as follows: score value below 1.699: the identification was unreliable; 1.700–1.999: probable genus identification; 2.000–2.299: reliable genus identification and probable species identification; and 2.300–3.000: highly probable species identification (23). The mass spectra were externally calibrated using the *Escherichia coli* DH5-alpha standard (Bruker Daltonics).

For the Biotyper database upgrading, the spectra of 16S rRNA-identified strains were incorporated. Running 24 replicates of each sample on MALDI-TOF MS, the spectra were analyzed by the Flex Analysis software. Low-intensity spectra were removed, and 20 good-quality records were used to create a reference Main Spectrum Profile (MSP) using the automated function of the Bruker Biotyper 3.1 software. The obtained MSPs have been implemented into the *in-house* MALDI-TOF MS database.

## RESULTS

### Strain isolation and morphology

The Actinobacteria strains isolated from building material samples originated mainly from samples with confirmed microbial growth. In all but one material sample, the microbial growth was confirmed by total fungal concentration above the limit value (10,000 cfu/g) or concentration of fungi moderately increased (5,000–10,000 cfu/g) plus the appearance of specific fungal or Actinobacteria moisture damage indicator taxa (9).

Regarding air samples used for the isolation of Actinobacteria*,* three indoor samples revealed a low or normal concentration of fungi (<100 cfu/m^3^), one sample had an increased concentration of fungi (100–500 cfu/m^3^) and occurrence of moisture damage indicator taxa, and two indoor samples had a high concentration of fungi (>500 cfu/m^3^) (9). One sample was collected from the outdoor air in the vicinity of a terraced house that contained no Actinobacteria in the indoor air sample.

The Actinobacteria isolates from building materials and air samples belonged to Gram-positive mesophilic bacteria with an optimal growth temperature of about 25°C. Most of the strains grew well on solid media, the white or grayish aerial mycelium appeared after 48–72 h, and the cultivation was continued for 10–14 days.

Few species produced a brown or violet diffusible pigment (Table S1; Fig. S1). Majority of isolates were filamented, sometimes branched rods (Fig. S2a, b, and e through l). Few strains identified later as *Pseudonocardia* produced shorted rods (Fig. S2c and d).

### Chemotaxonomic characteristics

As the isolates were supposed to be representatives of the *Streptomyces* genus due to colony morphology, cell wall component assessment, i.e., whole-cell DAP analysis, was performed. The majority of isolates revealed an L,L-isomer of DAP, except for seven species (AKT7, 8, 10, 13, 22, 24, and 26) that had meso-DAP (Table S1). To date, the I type of the cell wall with LL-DAP in peptidoglycan is a specific feature of *Streptomyces*. Polar lipid analysis revealed that all strains possess phosphatidylethanolamine, which is a taxonomic phospholipid indicating phospholipid type II (Table S1; Fig. S3b); phosphatidylcholine was found in three isolates (AKT8, 10, and 13) (Fig. S3a). Crude lipid analysis of the isolates revealed a lack of a significant amount of glycolipids; however, three different profiles could be distinguished: with one major glycolipid (g), with two glycolipids (2g), and without major glycolipids (Fig. S4; Table S1).

Mycolic acid (MA) analysis by TLC revealed the following MA content: AKT7, 22, 24, and 26 have mycolic acid with the same TLC mobility as nocardiomycolic acid suggesting that four isolates belong to the *Nocardia* genus (Fig. S5a and b). No difference in TLC mobility was observed between mycolic acids obtained by the acid and alkaline methods. The presence of N-glycolylated muramic acid in peptidoglycan was positively verified in AKT7, 22, 24, and 26 strains; in other isolates, N-glycolylation was not detected.

Whole-cell fatty acid (FA) analysis has been informative for strains AKT7, 22, 24, and 26 since they revealed a distinct fatty acyl profile than the majority of the strains studied: saturated fatty acids with one monounsaturated and 12-methylstearic acid (tuberculostearic acid) (Table S2). The rest of the strains possess a considerable amount of branched *iso* and *anteiso* C15:0, C16:0, and C17:0, which are typical for the *Streptomyces* genus (Table S2). Based on chemotaxonomic features, the majority of strains (21/28) were classified to *Streptomyces* genus and *Nocardia* genus (AKT7, 22, 24, and 26). The strains AKT8, 10, and 13 could not be successfully identified but were distinct from *Streptomyces* and did not contain mycolic acid, which excluded them from the *Corynebacterinae* suborder and *Nocardia* genus.

### 16S rRNA gene sequencing

**Fig 1:**
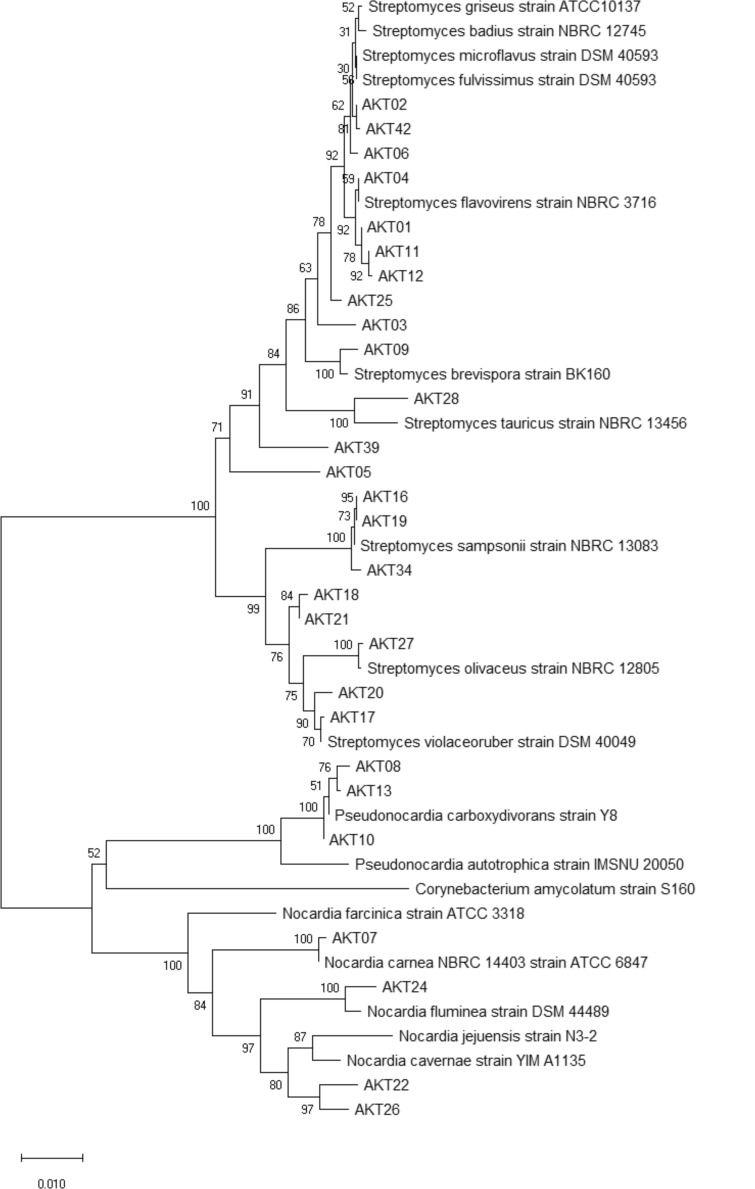
Neighbor-joining phylogenetic tree derived from the 16S rRNA sequences of Actinobacteria isolates and reference sequences. Evolutionary analyses were conducted in MEGA X (21).

Finally, all isolates were taxonomically allocated by 16S rRNA gene sequencing and alignment to the NCBI 16S ribosomal database (Table S3), largely with sequence identity values above 99%. The strains AKT8, 10, and 13, problematic in chemotaxonomic identification, turned out to be representatives of the *Pseudonocardia* genus (sequence similarity levels to *Pseudonocardia alni* >99%); isolates AKT7, 22, 24, and 26 were representatives of *Nocardia* genus (sequence similarity values between 98.04% and 99.85% to *Nocardia niigatenis, Nocardia mangyaensis, Nocardia cavernae,* and *N. carnea*). Most of the isolates (*n* = 21) were closely related to multiple *Streptomyces* species (most frequently *Streptomyces flavovirens, Streptomyces microflavus, Streptomyces alboviridis, Streptomyces flavogriseus, Streptomyces violaceolatus, Streptomyces coelicoflavus, Streptomyces sampsonii, Streptomyces coelicolor, Streptomyces limosus,* and *Streptomyces felleus*), with sequence similarity values largely >99%. Since, in many cases, several species showed the same sequence similarity values, unambiguous species-level taxonomic allocation was not feasible. The phylogenetic relationship of the strains is presented in Fig. 1.

### Identification of actinobacterial strains by MALDI-TOF MS

Preliminary experiments were performed to choose the best solid medium and cultivation time of building material isolates AKT1–AKT8 for reliable identification in the Bruker Biotyper system. Growth on the following media was evaluated: BL, NA, TS, yeast extract glucose agar (medium 79), and BHI during 2, 4, and 7 days at 25°C. The EFAE procedure recommended by the manufacturer was used in MALDI-TOF MS analysis. To date, identification on the genus level and the best score value in the MALDI-TOF MS analysis were gained using medium 79 and TS (Table S4). It is worth to underline that in the majority of cases, the strains were identified as *Streptomyces* spp. even when the score value was below 1.7 (indicating non-reliable identification).

In the next step, all actinobacterial isolates were identified in MALDI-TOF MS using different sample preparation methods: DT, DTFA, and in-tube EFAE in the same cultivation conditions (Table 2), i.e., medium 79 or TS according to preliminary experiments. Eleven isolates were identified mostly as *S. griseus, Streptomyces badius,* or *Streptomyces violaceoruber* with a score value above 1.7, indicating genus-level identification. One nocardial strain was identified as *Nocardia carnea* with a score value above 2.0. Unfortunately, the identification of the 16 strains was unreliable as the matching value was below 1.7 regardless of the sample preparation method used, i.e., AKT5, 8, 10, 11, 12, 13, 16, 19, 22, 24, 25, 26, 27, 28, 34, and 39 representing 57% of all isolates (Table 2). For the rest of the isolates surprisingly, utilizing different sample preparation methods revealed distinct results. The status of an identified sample using the EFAE procedure has been changed from not reliable to the reliable genus in six samples and five samples compared to DT and DTFA, respectively. Also, the mass spectra obtained by the extraction method contained more peaks than those analyzed by the direct transfer method (data not shown), which pointed to the extraction method as more reliable. Ultimately, using the EFAE method, the isolates AKT1, 2, 3, 17, 18, 20, and 21 were identified as *Streptomyces* sp. and AKT7 as *Nocardia carnea* with the highly reliable identification score. Interestingly, in four cases, the direct transfer methods (DT or DTFA) were significantly better than the extraction method (samples AKT4, 6, 9, and 42), providing that using solely the extraction method, the identification was unreliable. To date, comparing DT and DTFA, direct transfer with formic acid directly on the target plate was advantageous, and the higher identification score value was obtained in the case of 21 samples contrary to 7 samples.

**TABLE 2:** Identification of environmental isolates in MALDI-TOF MS database of samples obtained by the direct transfer methods and the extraction protocol^*b*^

No.	Sample/conditions	Identification result	Score value
1	AKT01_TS_48 h_DT	NR (*Streptomyces griseus*)^*a*^	1.408
	AKT01_TS_48 h_DTFA	*Streptomyces griseus*	1.955
	AKT01_TS_48 h_EFAE	*Streptomyces badius*	1.935
2	AKT02_TS_48 h_DT	*Streptomyces badius*	1.778
	AKT02_TS_48 h_DTFA	*Streptomyces badius*	1.919
	AKT02_TS_48 h_EFAE	*Streptomyces griseus*	1.716
3	AKT03_TS_48 h_DT	NR (*Streptomyces avidinii*)^*a*^	1.504
	AKT03_TS_48 h_DTFA	Not reliable	1.51
	AKT03_TS_48 h_EFAE	*Streptomyces badius*	1.756
4	AKT04_TS_48 h_DT	*Streptomyces badius*	2.038
	AKT04_TS_48 h_DTFA	*Streptomyces badius*	1.875
	AKT04_TS_48 h_EFAE	NR (*S. scabiei*)^*a*^	1.566
5	AKT05_TS_48h_DT	NR (*S. violaceoruber*)^*a*^	1.694
	AKT05_TS_48h_DTFA	NR (*S. albus*)^*a*^	1.508
	AKT05_TS_48 h_EFAE	Not reliable	1.366
6	AKT06_TS_48 h_DT	NR (*S. badius*)^*a*^	1.657
	AKT06_TS_48 h_DTFA	*Streptomyces badius*	1.727
	AKT06_TS_48 h_EFAE	NR (*S. griseus*)^*a*^	1.589
7	AKT07_TS_96 h_DT	*Nocardia carnea*	2.027
	AKT07_TS_96 h_DTFA	*Nocardia carnea*	2.331
	AKT07_TS_96 h_EFAE	*Nocardia carnea*	2.326
8	AKT08_79_72 h_DT	Not reliable	1.428
	AKT08_79_72 h_DTFA	Not reliable	1.49
	AKT08_79_48 h_EFAE	Not reliable	1.373
9	AKT09_79_72 h_DT	*Streptomyces griseus*	1.977
	AKT09_79_72 h_DTFA	*Streptomyces badius*	1.844
	AKT09_79_48 h_EFAE	NR (*S. griseus*)^*a*^	1.536
10	AKT10_79_72 h_DT	Not reliable	1.398
	AKT10_79_72 h_DTFA	Not reliable	1.403
	AKT10_79_48 h_EFAE	Not reliable	1.248
11	AKT11_79_72 h_DT	NR (*S. badius*)^*a*^	1.486
	AKT11_79_72 h_DTFA	NR (*S. phaeochromogenes*)^*a*^	1.509
	AKT11_79_48 h_EFAE	NR (*S. badius*)^*a*^	1.465
12	AKT12_79_72 h_DT	NR (*S. griseus*)^*a*^	1.43
	AKT12_79_72 h_DTFA	NR (*S. scabiei*)^*a*^	1.678
	AKT12_79_48 h_EFAE	NR (*S. badius*)^*a*^	1.419
13	AKT13_79_48 h_DT	Not reliable	1.469
	AKT13_79_48 h_DTFA	NR (*S. phaeochromogenes*)^*a*^	1.468
	AKT13_79_48 h_EFAE	Not reliable	1.494
14	AKT16_79_48 h_DT	Not reliable	1.411
	AKT16_79_48 h_DTFA	Not reliable	1.452
	AKT16_79_72 h_EFAE	Not reliable	1.415
15	AKT17_79_72 h_DT	Not reliable	1.349
	AKT17_79_72 h_DTFA	Not reliable	1.451
	AKT17_79_72 h_EFAE	*Streptomyces violaceoruber*	1.8
16	AKT18_79_72 h_DT	NR (*S. lavendulae*)^*a*^	1.489
	AKT18_79_72 h_DTFA	Not reliable	1.575
	AKT18_79_72 h_EFAE	*Streptomyces violaceoruber*	1.721
17	AKT19_79_72 h_DT	Not reliable	1.509
	AKT19_79_48 h_DTFA	NR (*S. badius*)^*a*^	1.461
	AKT19_79_72 h_EFAE	Not reliable	1.242
18	AKT20_79_72 h_DT	Not reliable	1.649
	AKT20_79_72 h_DTFA	NR (*S. violaceoruber*)^*a*^	1.586
	AKT20_79_72 h_EFAE	*Streptomyces violaceoruber*	1.849
19	AKT21_79_72 h_DT	NR (*S. violaceoruber*)^*a*^	1.465
	AKT21_79_72 h_DTFA	Not reliable	1.494
	AKT21_79_72 h_EFAE	*Streptomyces violaceoruber*	1.826
20	AKT22_79_72h_DT	Not reliable	1.464
	AKT22_79_72h_DTFA	Not reliable	1.374
	AKT22_79_48h_EFAE	Not reliable	1.455
21	AKT24_79_72 h_DT	NR (*S. badius*)^*a*^	1.418
	AKT24_79_72 h_DTFA	Not reliable	1.462
	AKT24_79_48 h_EFAE	Not reliable	1.377
22	AKT25_79_72 h_DT	Not reliable	1.244
	AKT25_79_72 h_DTFA	Not reliable	1.205
	AKT25_79_48 h_EFAE	Not reliable	1.234
23	AKT26_79_48 h_DT	Not reliable	1.382
	AKT26_79_48 h_DTFA	Not reliable	1.424
	AKT26_79_48 h_EFAE	Not reliable	1.305
24	AKT27_79_72 h_DT	Not reliable	1.389
	AKT27_79_72 h_DTFA	Not reliable	1.563
	AKT27_79_72 h_EFAE	Not reliable	1.307
25	AKT28_79_72 h_DT	Not reliable	1.508
	AKT28_79_72 h_DTFA	NR (*S. avidinii*)^*a*^	1.61
	AKT28_79_72 h_EFAE	Not reliable	1.302
26	AKT34_79_48 h_DT	NR (*S. chartreusis*)^*a*^	1.522
	AKT34_79_48 h_DTFA	NR (*S. violaceoruber*)^*a*^	1.692
	AKT34_79_72 h_EFAE	Not reliable	1.365
27	AKT39_79_72 h_DT	Not reliable	1.44
	AKT39_79_48 h_DTFA	NR (*S. phaeochromogenes*)^*a*^	1.526
	AKT39_79_72 h_EFAE	Not reliable	1.398
28	AKT42_79_48 h_DT	*Streptomyces griseus*	2.001
	AKT42_79_48 h_DTFA	*Streptomyces griseus*	1.961
	AKT42_79_72 h_EFAE	Not reliable	1.297

^*a*^ NR, not reliable identification, score value below 1.7, in parentheses best-match identification.

^*b*^ Abbreviations: isolates were cultivated on TS or yeast extract glucose agar (79 medium) for 48–96 h at 26°C; DT, direct transfer; DTFA, direct transfer method with formic acid treatment on the target plate; EFAE, ethanol-formic acid extraction procedure.

### MALDI-TOF MS database upgrading

In an effort to improve the performance of MALDI-TOF MS in the identification of Actinobacteria isolates, we upgraded the in-house MALDI-TOF MS database with eight isolates: AKT1 *S. flavovirens*, AKT2 *S. microflavus*, AKT10 *Pseudonocardia alni*, AKT17 *S. violaceolatus*, AKT19 *S. sampsonii*, AKT21 *S. coelicoflavus*, AKT24 *Nocardia mangyaensis,* and AKT26 *N. cavernae*. In Table S5, the best score value obtained in Bruker Biotyper analysis is compared with the result of identification in the in-house database. After re-evaluation of mass spectra with an in-house database, 13 additional species improved the score value from “not reliable” to at least “probable genus,” i.e., AKT5, 8, 10, 11, 12, 13, 16, 19, 22, 24, 26, 27, and 34 (Table S5; Table 3). Three *Streptomyces* isolates (AKT25, 28, and 39) remained not reliably identified by the Biotyper database and upgraded MALDI-TOF MS in-house database (Table S5).

**TABLE 3:** Comparison of identification results based on chemotaxonomy, 16S rRNA, and MALDI-TOF MS *in-house* database

AKT no.	Genus based on chemotaxonomy	Closest database match species based on 16S rRNA gene sequencing	% sequence similarity to the database entry	Identification in-house MALDI-TOF MS database	Score value
AKT01	*Streptomyces*	*Streptomyces flavovirens, S. flavogriseus*	100%	*Streptomyces flavovirens*	2.290
AKT02	*Streptomyces*	*Streptomyces microflavus, S. alboviridis*	99.85%	*Streptomyces microflavus*	2.259
AKT03	*Streptomyces*	*Streptomyces sanglieri*	99.09%	*Streptomyces flavovirens*	1.776
AKT04	*Streptomyces*	*Streptomyces flavovirens, S. flavogriseus*	99.93%	*Streptomyces flavovirens*	1.982
AKT05	*Streptomyces*	*Streptomyces albiaxialis*	99.18%	*Streptomyces microflavus*	2.185
AKT06	*Streptomyces*	*Streptomyces microflavus, S. alboviridis*	99.78%	*Streptomyces microflavus*	2.185
AKT07	*Nocardia*	*Nocardia carnea*	99.85%	NR/*Nocardia cavernae*	1.482
AKT08	unidentified	*Pseudonocardia alni*	99.56%	*Pseudonocardia alni*	2.075
AKT09	*Streptomyces*	*Streptomyces brevispora*	99.42%	NR/*Streptomyces microflavus*	1.179
AKT10	unidentified	*Pseudonocardia alni*	100%	*Pseudonocardia alni*	2.333
AKT11	*Streptomyces*	*Streptomyces flavovirens, S. flavogriseus*	99.93%	*Streptomyces flavovirens*	1.903
AKT12	*Streptomyces*	*Streptomyces flavovirens, S. flavogriseus*	99.86%	*Streptomyces flavovirens*	1.957
AKT13	unidentified	*Pseudonocardia alni*	99.71%	*Pseudonocardia alni*	1.851
AKT16	*Streptomyces*	*Streptomyces sampsonii, Streptomyces hydrogenans, S. coelicolor, S. limosus, S. felleus*	99.93%	*Streptomyces sampsonii*	1.826
AKT17	*Streptomyces*	*Streptomyces violaceolatus*	100%	*Streptomyces violaceolatus*	2.415
AKT18	*Streptomyces*	*Streptomyces coelicoflavus*	99.85%	*Streptomyces coelicoflavus*	2.041
AKT19	*Streptomyces*	*Streptomyces sampsonii, S. hydrogenans, S. coelicolor, S. limosus, S. felleus*	100%	*Streptomyces sampsonii*	1.844
AKT20	*Streptomyces*	*Streptomyces violaceolatus*	99.49%	*Streptomyces violaceolatus*	2.379
AKT21	*Streptomyces*	*Streptomyces coelicoflavus*	100%	*Streptomyces coelicoflavus*	2.133
AKT22	*Nocardia*	*Nocardia niigatensis*	98.04%	*Nocardia cavernae*	1.864
AKT24	*Nocardia*	*Nocardia mangyaensis*	99.20%	*Nocardia mangyaensis*	1.829
AKT25	*Streptomyces*	*Streptomyces griseobrunneus, S. cavourensis, S. bacillaris, Kitasatospora albolonga*	99.53%	NR/*Streptomyces microflavus*	1.047
AKT26	*Nocardia*	*Nocardia cavernae*	98.15%	*Nocardia cavernae*	1.815
AKT27	*Streptomyces*	*Streptomyces olivaceus, S. pactum*	99.71%	*Streptomyces violaceolatus*	1.773
AKT28	*Streptomyces*	*Streptomyces songpinggouensis, Streptomyces tauricus*	98.45%	NR/*Streptomyces microflavus*	1.034
AKT34	*Streptomyces*	*Streptomyces resistomycificus, S. hydrogenans, S. sampsonii, S. coelicolor, S. limosus, S. felleus, Streptomyces griseochromogenes*	99.78%	*Streptomyces sampsonii*	2.125
AKT39	*Streptomyces*	*Streptomyces abietis*	98.55%	NR/*Streptomyces flavovirens*	1.015
AKT42	*Streptomyces*	*Streptomyces microflavus, S. alboviridis*	99.86%	*Streptomyces microflavus*	2.187

In Table 3, the identification results obtained using chemotaxonomy, 16S rRNA gene sequencing, and MALDI-TOF MS in-house database of AKT isolates were compared.

## DISCUSSION

In this study, we set out to meet the challenge of identification of (indoor) environmental Actinobacteria isolates and compared chemotaxonomic/morphological characterization and 16S rRNA gene sequencing to a MALDI-TOF MS-based method. The latter has the potential to be a rapid, less laborious approach to species-level identification, compared to the polyphasic, labor-intensive, chemotaxonomic/morphological approach or 16S rRNA gene sequencing that suffers from limited species-level discriminatory power for specific taxonomic groups, including *Streptomyces* (24). Our study confirms the potential of MALDI-TOF MS in environmental strain identification but also highlights the need to build custom-made databases for the target species to improve the taxonomic resolution of the method.

The motivation for this work was twofold: one, robust and fast identification of Actinobacteria isolates from building materials, house dust, or indoor air could potentially improve the value of microbial measurements in moisture-damaged building investigations. It is well known that Actinobacteria taxa of certain genera can grow together with fungi on wet building materials and, thus, indicate moisture problems (6, 7). At the same time, Actinobacteria are ubiquitous in our environment and occur in soil, water, and outdoor air (5), so that their occurrence indoors could also reflect other sources than moisture damage. A method that would more accurately speciate Actinobacteria from indoor samples and allow for a more accurate source allocation could help the interpretation of indoor microbial measurements in the context of building inspections (25). Two, the contribution of microbial taxa, including *Streptomyces* and other Actinobacteria taxa, to the adverse health effects observed in occupants of moisture-damaged buildings is not well understood (11, 26, 27). There is a consistent suggestion from toxicological *in vitro* and *in vivo* studies that microbes, specifically also *Streptomyces* species, may contribute to the adverse health effects observed in occupants of damp buildings (26, 28–31). However, since this earlier work, the few epidemiological studies investigating associations between indoor exposure to moisture damage-related Actinobacteria and adverse health effects have failed to present consistent and strong support for the health relevance of *Streptomyces* or other Actinobacteria genera indoors (32–37). More specific characterization of indoor *Streptomyces* and other moisture damage-related Actinobacteria taxa could be valuable to efforts aiming at clarifying the health relevance of specific bacterial groups.

The identification of clinical as well as environmental strains of Actinobacteria at the species level is complex and challenging. Historically, morphology and biochemical approaches as well as chemotaxonomic methods were developed preceding gene-based identification. *Nocardia* and *Pseudonocardia* species represent cell wall type IV with meso-DAP, Ara, and Gal in the cell wall (38), in contrast to *Streptomyces* spp. belonging to cell wall chemotype I (39). On the other hand, N-acetylmuramic acid was found in the cell wall of *Streptomyces*, as in most actinomycetes (40) with the exception of *Nocardia,* which is of N-glycolylmuramic acid type. Also, fatty acids and polar lipids represent potential as taxonomic markers, useful in discrimination on the genus level. Chemotaxonomy as a part of polyphasic taxonomy is an important tool for novel species description (41). Notably, such an approach is complicated, laborious, time-consuming, and not accessible in many laboratories.

In our work, the chemotaxonomic characteristics of actinobacterial strains isolated from building materials as well as indoor and outdoor air were performed, and chemical markers have been determined to provide identification on the genus level. Representatives of *Streptomyces* and *Nocardia* were found. The most informative were DAP and mycolic acid analyses (Table S1).

Methods based on genomic analysis, such as DNA–DNA hybridization, 16S ribosomal RNA gene sequence, and whole genome sequencing, are well-established additions to bacterial taxonomy studies. The 16S ribosomal RNA gene is an efficient molecular marker, considered universal, functionally stable, highly conserved, and persistent to horizontal gene transfer (42). However, the resolving power of 16S rRNA sequences is not sufficient to differentiate species within the same genus, as, for example, in the *Streptomyces* genus caused mainly by the heterogeneity among different 16S rRNA gene copies within the genome (24, 43). The results of our study certainly confirm this early reported concern. We were able to match our indoor isolates to *Streptomyces*, *Nocardia,* and *Pseudonocardia* strains of a 16S rRNA gene sequence reference database at high sequence similarity values (largely >99%). However, in many cases, several different *Streptomyces* species matched our isolates at the same similarity percentage, making an unambiguous species-level allocation based on 16S rRNA gene sequencing impossible (Table 3).

The proteomic methods based on mass spectrometry are promising and rapidly complement or replace traditional methods of bacterial identification. MALDI-TOF mass spectrometry is reliable, fast and relatively inexpensive, and, therefore, widely applied in clinical microbiology (44). The available information on the potential and challenges of expanding MALDI-TOF MS into microbiological ecology studies has recently been reviewed (45). The inappropriate identification of environmental isolates by MALDI-TOF mass spectrometry is caused mainly by database content. In the present studies, we used the Biotyper 3.1 database containing 6,904 entries. The *Streptomyces* genus comprises about 600 species (http://www.bacterio.net/index.html) while our MALDI-TOF Biotyper database contained only 17 mass spectra for 14 reference *Streptomyces* species. Moreover, the *Nocardia* genus containing about 100 species was represented by 105 *Nocardia* mass spectra referred to 38 species, and the *Pseudonocardia* genus containing about 60 species was represented only by one species.

MALDI-TOF MS analysis of 28 environmental isolates utilizing the commercial Bruker database enabled the identification of only 12 isolates (Table 2). The unsatisfactory results of *Streptomyces* isolate identification by MALDI-TOF MS have been also noted by other authors (46, 47) but could be partly overcome by in-house upgrading of the database (46, 48). In this work, after upgrading the in-house database with just eight strains, we were able to identify 13 additional isolates (Table S5), so that 25/28 isolates could be identified.

To obtain reliable bacterial strain identification by MALDI-TOF MS, the appropriate sample preparation is important. In general, three different methods can be used: direct sample spotting (DT), on-target extraction (DTFA), and in-tube extraction procedure (EFAE). The simplest and fastest DT is frequently used in clinical laboratories. This method is, however, not recommended for some Gram-positive bacteria (for ex., *Actinomyces* and *Nocardia*) and *Mycobacterium*. Wang et al. found the DTFA method as the best procedure for routine clinical microbiology due to its simplicity and accuracy (49). However, the limitation of this study was an elaboration of common clinical strains, and a small number of *Mycobacterium* species, also filamentous fungi, were not included.

The extraction procedure has few steps and takes longer than direct methods but is used for difficult-to-identify microorganisms (50). It was estimated that for the extraction procedure, approx. 10^6^–10^7^ cells are needed (51). Due to the thickness and hydrophobicity of the actinobacterial cell walls, in MALDI-TOF MS analysis, the extraction method is preferred. We tried to improve MALDI-TOF MS identification by using different sample preparation methods. We observed that more species were identified at the genus level using the in-tube EFAE procedure instead of the direct colony transfer method; however, in four cases, the direct methods provided better results (Table 2). The EFAE extraction method is a longer procedure; nonetheless, we recommended using both the EFAE and DTFA.

Comparing chemotaxonomic methods and MALDI-TOF MS analysis with 16S rRNA gene sequencing results, it is worth stressing that in this work, we have identified all 21 *Streptomyces* species on the genus level using chemotaxonomy, contrary to 11 species identified by MALDI-TOF MS prior to the database improvement. In the case of *Nocardia* isolates, four species were identified on the genus level by chemotaxonomy (Table 3) and one by MALDI-TOF MS on the species level. Summing up, the MALDI-TOF MS technique is a very fast and good alternative for rapid screening; although in cases where identification is not reliable, it can be recommended to rely on traditional, trusted chemotaxonomic methods.

### Conclusions

MALDI-TOF MS has a high potential in environmental strain identification; nevertheless, in the case of environmental Actinobacteria, the database used needs to contain significantly more environmental Actinobacteria representatives. This technique proved to be excellent for the fast screening of isolates, and in case of doubtful identification according to availabilities, it can be solved by 16S rRNA gene sequencing or even by chemotaxonomy.

## Data Availability

All data generated or analyzed during this study are included in this published article and its supplemental material. The actinobacterial strains AKT01–AKT42 originally isolated from building materials and air samples in Finland were deposited in the Polish Collection of Microorganisms (PCM) (Table 1). The nucleotide sequences of 16S rRNA were deposited in GenBank (Table S3).

## SUPPLEMENTAL MATERIAL

Supplemental material spectrum.03596-23-s0001.docx Fig. S1 to S5; Tables S1 to S5.

ASM does not own the copyrights to Supplemental Material that may be linked to, or accessed through, an article. The authors have granted ASM a non-exclusive, world-wide license to publish the Supplemental Material files. Please contact the corresponding author directly for reuse.
