# Supplementary material for: Identification of environmental Actinobacteria in buildings by means of chemotaxonomy, 16S rRNA sequencing, and MALDI-TOF MS
Source: Microbiol Spectr. 2024 Feb 1;12(3):e03596-23. doi: 10.1128/spectrum.03596-23 (PMC10913483; doi:10.1128/spectrum.03596-23)
Supplement: Supplemental material — Fig. S1 to S5; Tables S1 to S5. [file spectrum.03596-23-s0001.docx]

**Identification of environmental Actinobacteria in buildings by means of chemotaxonomy, 16S rRNA sequencing, and MALDI-TOF MS**

Anna Chudzik^1^, Kaisa Jalkanen^2^, Martin Täubel^2^, Bogumiła Szponar^1^, Mariola Paściak^1^*

^1^ Hirszfeld Institute of Immunology and Experimental Therapy, Polish Academy of Sciences, Rudolfa Weigla 12, Wroclaw, Poland

^2^ Environmental Health Unit, Finnish Institute for Health and Welfare, P.O. Box 95, Kuopio 70701, Finland

*Correspondence: mariola.pasciak@hirszfeld.pl

**Supplementary Information**

1. AKT 01_NA_72h b) AKT 03_NA_7 days


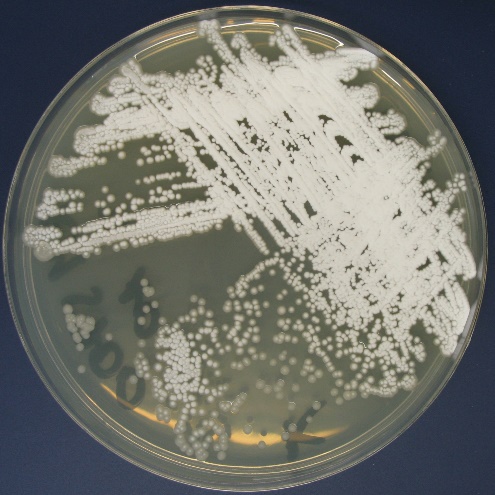

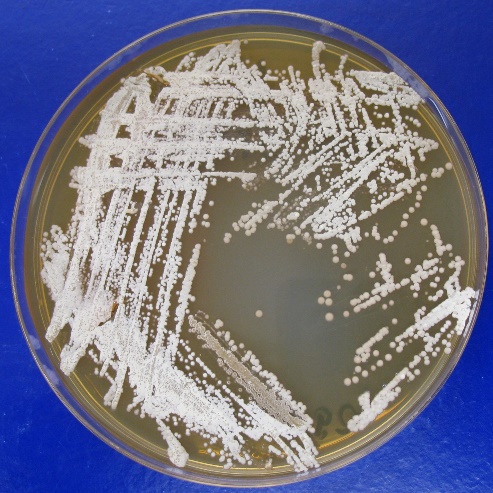


c) AKT 04_NA_7 days d) AKT 05_79_7 days


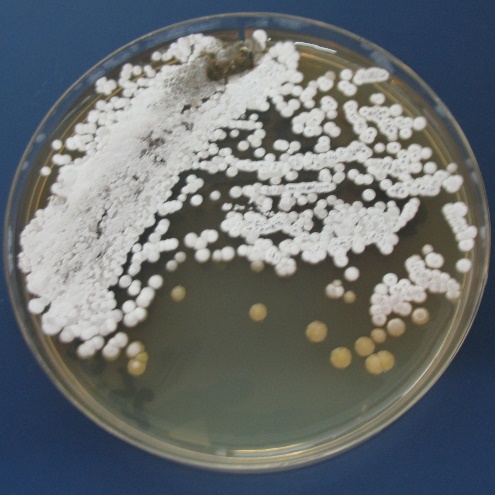

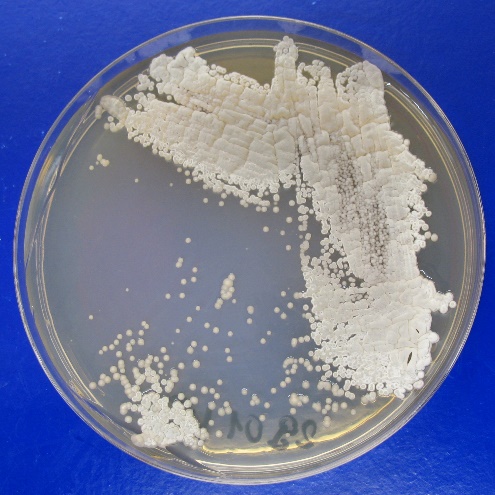


e) AKT 05_79_7 days f) AKT 06_79_4 days


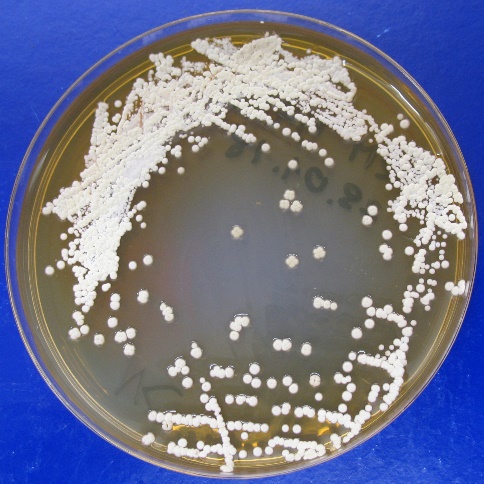

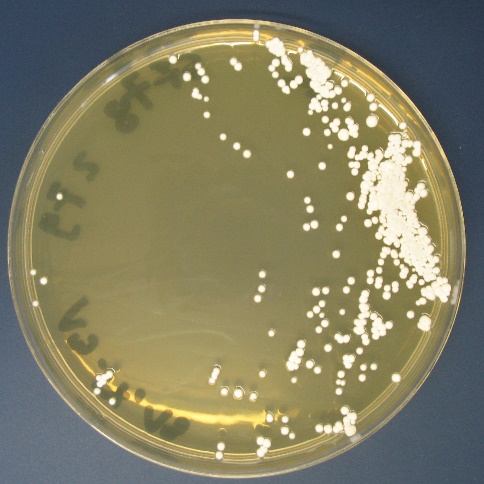


g) AKT 07_79_7 days h) AKT 07_NA_7 days


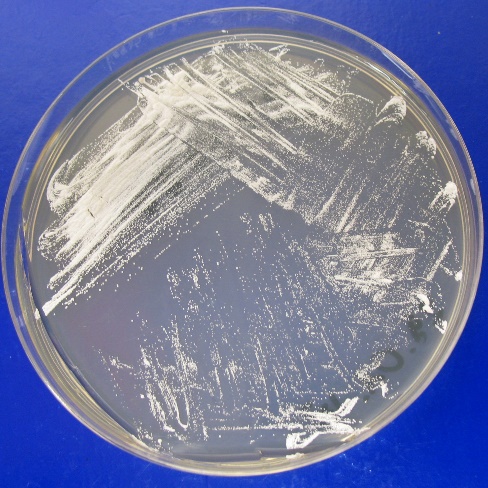

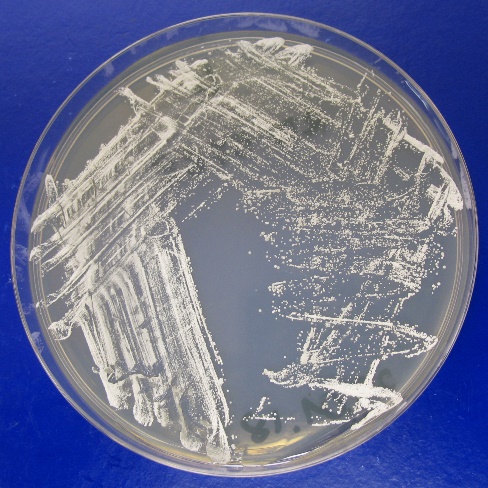


i) AKT 13_BL_48 h j) AKT 13_79_48 h


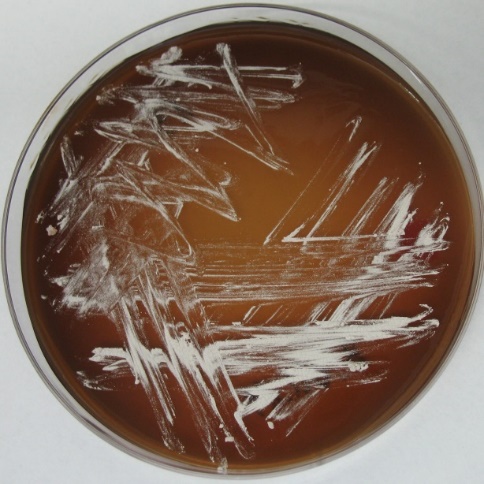

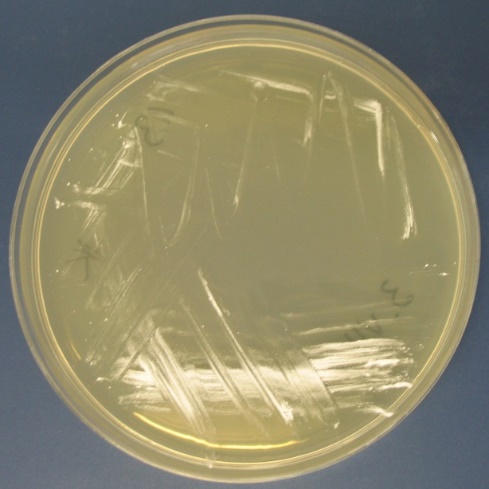


k) AKT 17_NA_10 days l) AKT 17_NA_10 days


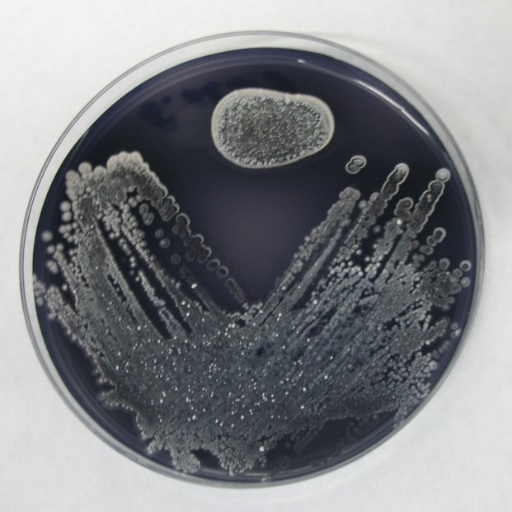

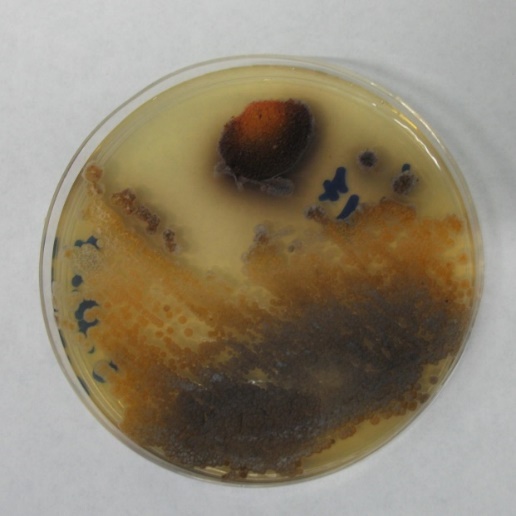


m) AKT 28_NA_72 h n) AKT 28_79_72 h


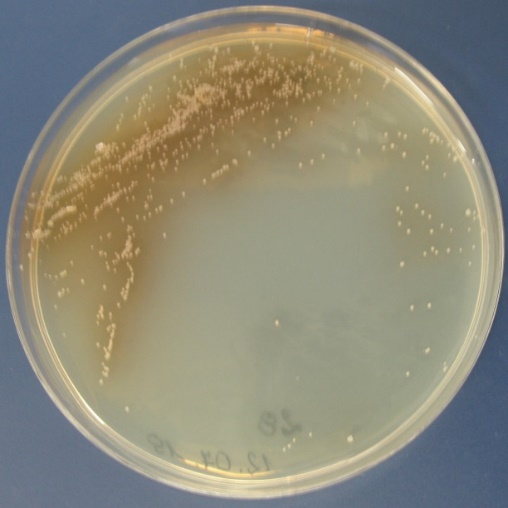

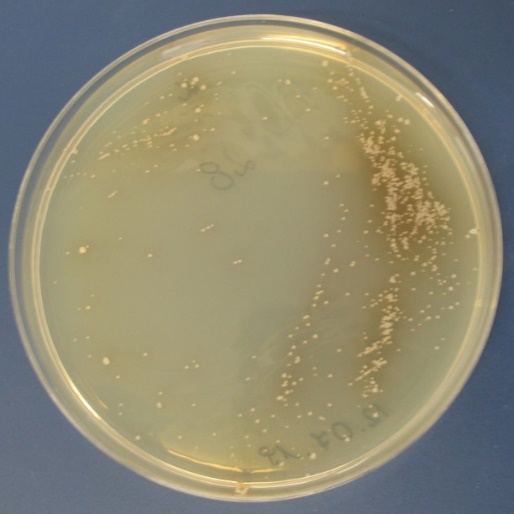


o) AKT 34_BL_72 h p) AKT 34_79_72 h


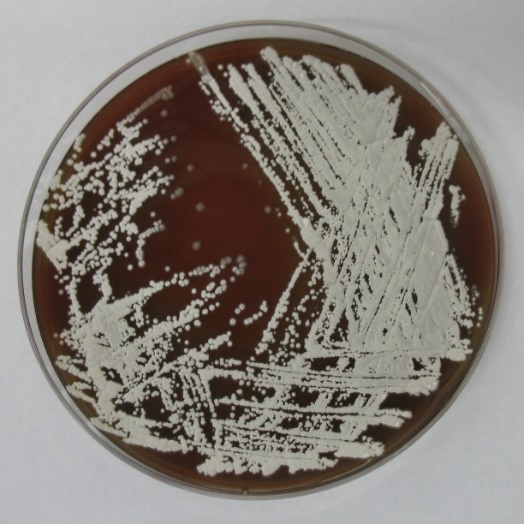

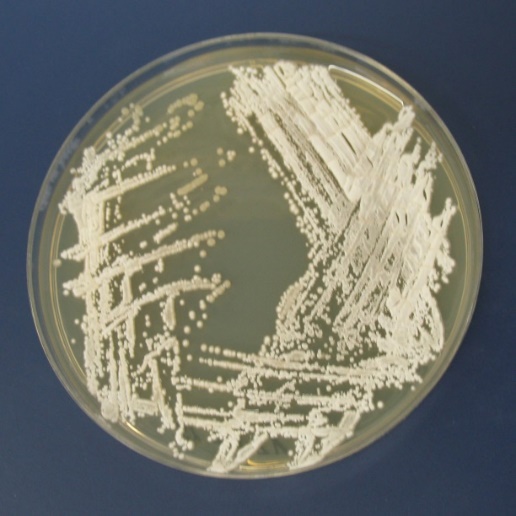


**Fig. S1**. Colony morphology of actinobacterial isolates grown on different media: NA, nutrient agar, BL, blood agar (nutrient agar with 5% sheep blood), 79, yeast–extract glucose agar, at 26°C for 48h -10 days.

| Blood agar | Yeast-extract glucose agar |
| --- | --- |
| a) AKT 12  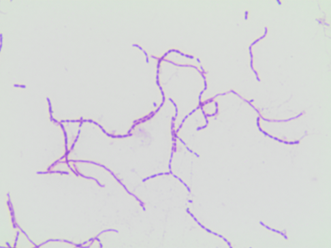 | b) AKT 12  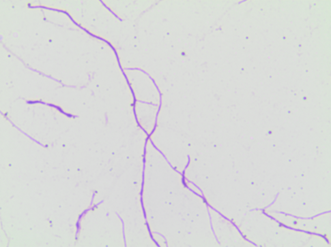 |
| c) AKT 13  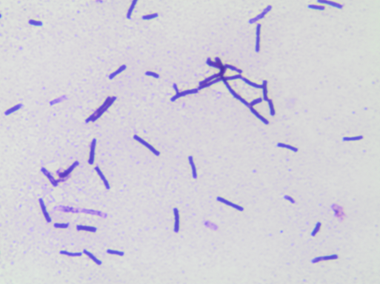 | d) AKT 13  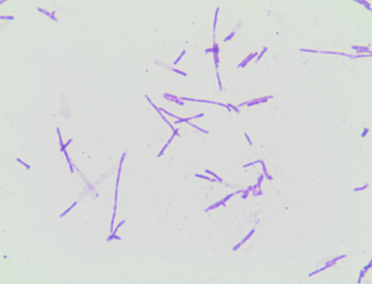 |
| e) AKT 21  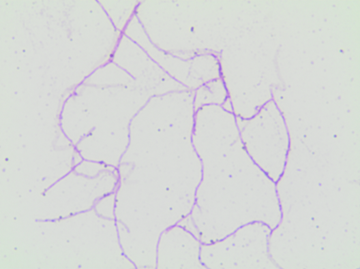 | f) AKT 21  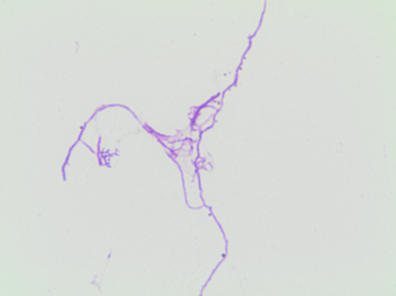 |
|  |  |
| g) AKT 27  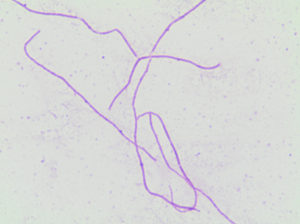 | h) AKT 27  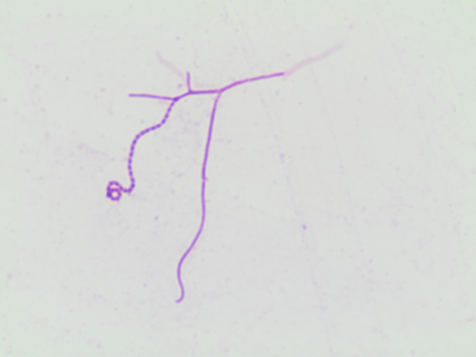 |
| i) AKT 34  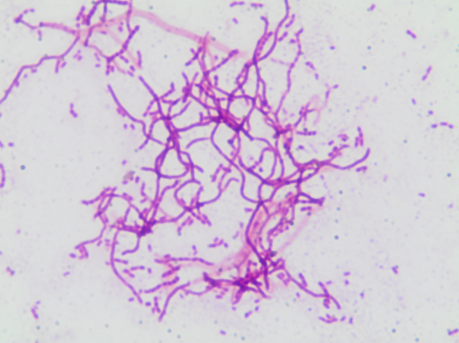 | j) AKT 34  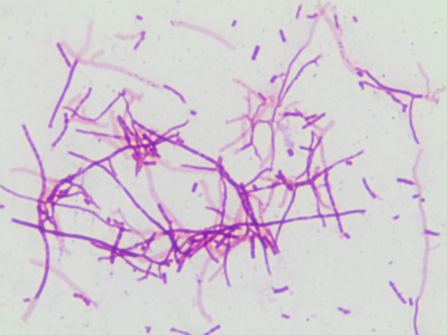 |
| k) AKT 42  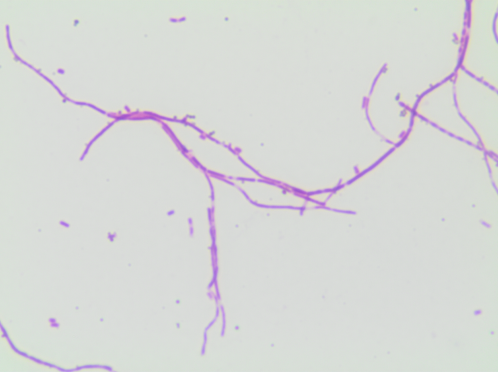 | l) AKT 42  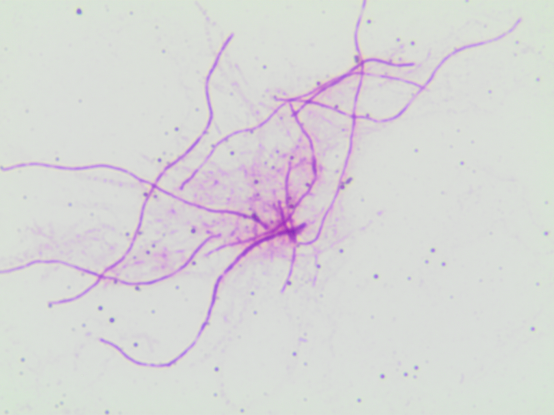 |
|  |  |

**Fig. S2.** Cell morphology of building material and air sample isolates cultivated on different solid media: a) AKT 12_BL, b) AKT 12_79, c) AKT 13_BL, d) AKT 13_79, e) AKT 21_BL, f) AKT 21_79, g) AKT 27_BL, h) AKT 27_79, i) AKT 34_BL, j) AKT 34_79, k) AKT 42_BL, l) AKT 42_79. The strains AKT 11, 13, 22, 42 were cultivated on blood agar (BL) and yeast-extract glucose agar (medium 79) for 48h at 28°C and strains AKT 28, 34 in the same conditions but for 72h; Gram staining, light microscopy x 1,500. Many AKT strains in young cultures tended to discolour in the Gram stain reaction.

a) b)


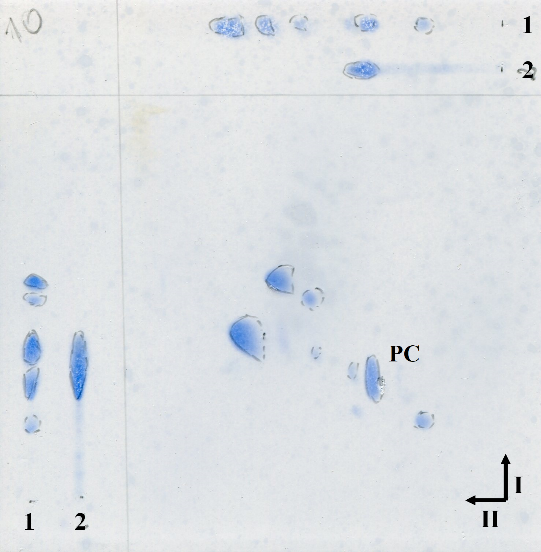

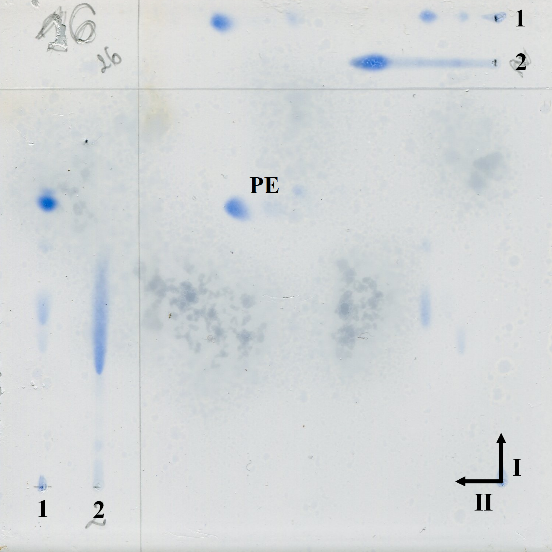


**Fig. S3**. 2D TLC of phospholipid profiles of AKT 10 (**a)** and AKT 26 (**b**); 1, lipid extract, 2, phosphatidylcholine standard (PC). Solvent systems (I) chloroform – methanol-water (65:25:4, v/v/v), (II) chloroform - acetic acid – methanol-water 80:15:12:4, v/v/v/v). Detection - Ditmer and Lester's reagent.

**
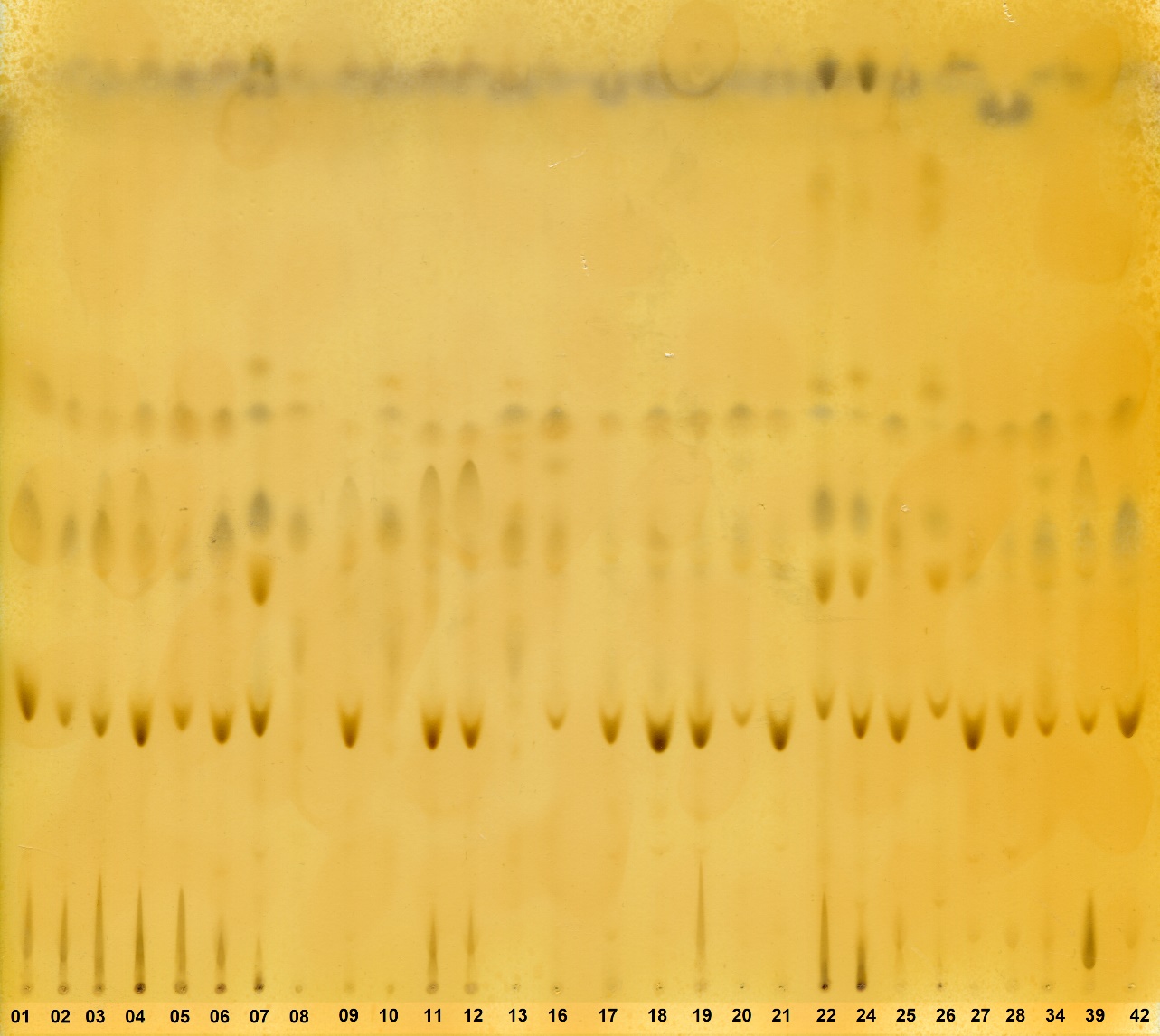
Fig. S4**. Glycolipid profile of building material and air sample isolates: AKT 01,02, 03, 04, 05, 06, 07, 08, 09, 10, 11, 12, 13, 16, 17, 18, 19, 20, 21, 22, 24, 25, 26, 27, 28, 34, 39, 42. Solvent system: chloroform-methanol-water (65:25:4 v/v/v), detection - orcinol reagent. Glycolipids are marked with arrows.

a) b)

| 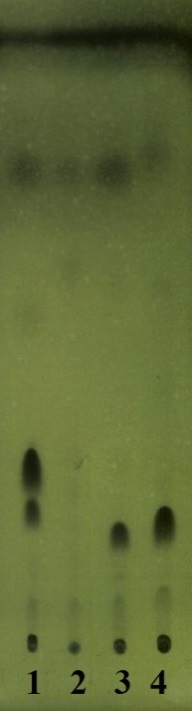 | 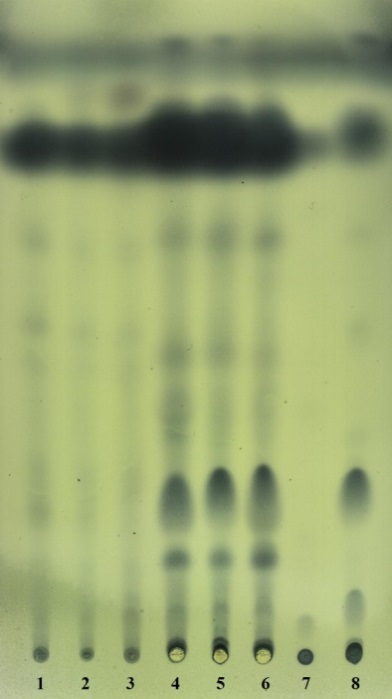 |
| --- | --- |

**Fig. S5.** Mycolic acids methyl esters (MAMES) of building material and air sample isolates. a) MAMES obtained by the alkaline method: 1. *T. paurometabola* PCM 2453, 2. *S. griseus* PCM 2331, 3. AKT 07, 4. *N. farcinica* PCM 2712. b) MAMES obtained by acid hydrolysis: 1. AKT 08, 2. AKT 10, 3. AKT 13, 4. AKT 22, 5. AKT 24, 6. AKT 26, 7. AKT 25, 8. *N. farcinica* PCM 2712. Solvent system: hexane-diethyl ether (85:15, v/v). Detection: molybdophosphoric acid (10% [w/v] in 95% [v/v] ethanol). Mycolic acid methyl esters are marked with arrows.

**Table S1** Morphologic and chemotaxonomic characteristics of the AKT isolates

|  | Reverse colour on 79 medium | Colour of aerial mycelium | Diffusible pigment | DAP isomer | Phospholipid type | Glycolipid  profile | Branched fatty acids | Tuberculo-stearic acid | Mycolic acids | Genus  based on chemotaxonomy |
| --- | --- | --- | --- | --- | --- | --- | --- | --- | --- | --- |
| AKT01 | yellow-orange | white |  | LL | II | g | + | - | - | *Streptomyces* |
| AKT02 | yellow | white |  | LL | II | g | + | - | - | *Streptomyces* |
| AKT03 | yellow-orange | white-gray | brown | LL | II | g | + | - | - | *Streptomyces* |
| AKT04 | orange-gray | white |  | LL | II | g | + | - | - | *Streptomyces* |
| AKT05 | yellow | white |  | LL | II | g | + | - | - | *Streptomyces* |
| AKT06 | yellow | white |  | LL | II | g | + | - | - | *Streptomyces* |
| AKT07 | yellow-orange | white |  | meso | II | 2g | - | + | + | *Nocardia* |
| AKT08 | yellow-pink | white-pink |  | meso | III | - | + | - | - | unidentified |
| AKT09 | yellow | gray | brown | LL | II | g | + | - | - | *Streptomyces* |
| AKT10 | yellow-orange | white-pink |  | meso | III | - | + | - | - | unidentified |
| AKT11 | yellow | white-gray |  | LL | II | g | + | - | - | *Streptomyces* |
| AKT12 | cream | white-gray |  | LL | II | g | + | - | - | *Streptomyces* |
| AKT13 | yellow-orange | white-pink |  | meso | III | - | + | - | - | unidentified |
| AKT16 | yellow | white |  | LL | II | g | + | - | - | *Streptomyces* |
| AKT17 | yellow | white-pink | violet | LL | II | g | + | - | - | *Streptomyces* |
| AKT18 | orange | white-pink |  | LL | II | g | + | - | - | *Streptomyces* |
| AKT19 | yellow | white |  | LL | II | g | + | - | - | *Streptomyces* |
| AKT20 | yellow | cream | violet | LL | II | g | + | - | - | *Streptomyces* |
| AKT21 | orange-red | pink |  | LL | II | g | + | - | - | *Streptomyces* |
| AKT22 | yellow-orange | white-pink |  | meso | II | 2g | - | + | + | *Nocardia* |
| AKT24 | yellow-orange | white |  | meso | II | 2g | - | + | + | *Nocardia* |
| AKT25 | yellow-orange | white |  | LL | II | g | + | - | - | *Streptomyces* |
| AKT26 | yellow-orange | white |  | meso | II | 2g | - | + | + | *Nocardia* |
| AKT27 | cream | white |  | LL | II | g | + | - | - | *Streptomyces* |
| AKT28 | yellow | white | brown | LL | II | g | + | - | - | *Streptomyces* |
| AKT34 | yellow | white-gray |  | LL | II | g | + | - | - | *Streptomyces* |
| AKT39 | yellow | cream |  | LL | II | g | + | - | - | *Streptomyces* |
| AKT42 | yellow | white |  | LL | II | g | + | - | - | *Streptomyces* |

**Table S2** Whole-cell fatty acid (FA) analysis of building material and air sample isolates; % of total fatty acids

|  | |  |  |  |  |  |  |  |  |  |  |  |  |  |  |  |  |  |  |
| --- | --- | --- | --- | --- | --- | --- | --- | --- | --- | --- | --- | --- | --- | --- | --- | --- | --- | --- | --- |
| AKT no | n 13:0 | i 14:0 | n 14:0 | i 15:0 | ai 15:0 | n 15:0 | 16:1* | i 16:0 | 16:1 (9) | n 16:0 | br 16:0 | i 17:0 | ai 17:0 | n 17:0 | 18:1* | 18:1 (9) | n 18:0 | TBS | n 20:1 |
| 1 |  | 1.44 |  | 4.82 | 38.36 | 1.17 |  | 17.14 | 1.08 | 4.87 | 1.37 | 4.5 | 24.14 | 0.78 |  |  | 0.38 |  |  |
| 2 |  | 1.18 |  | 3.57 | 41.54 | 1.16 |  | 21.34 | 2.05 | 2.88 | 1.36 | 2.23 | 21.99 | 0.43 |  |  | 0.26 |  |  |
| 3 |  | 0.38 |  | 4.83 | 49.37 | 0.94 |  | 6.19 | 2.66 | 7.51 | 1.39 | 2.38 | 23.71 | 0.4 |  |  | 0.25 |  |  |
| 4 |  | 0.96 |  | 5.37 | 38.02 | 1.01 |  | 13.31 | 1.48 | 8.66 | 1.7 | 4.93 | 23.12 | 0.76 |  |  | 0.67 |  |  |
| 5 |  | 1.06 |  | 8.33 | 31.26 | 0.62 |  | 33.31 | 0.59 | 1.67 | 0.56 | 3.36 | 18.43 | 0.56 |  |  | 0.24 |  |  |
| 6 |  | 1.22 |  | 2.18 | 37.25 | 1.29 |  | 23.24 | 1.64 | 2.38 | 1.13 | 1.81 | 26.74 | 0.68 |  |  | 0.44 |  |  |
| 7 |  |  | 0.42 |  |  |  |  |  | 7.91 | 27.47 |  |  |  |  |  | 40.17 | 6.47 | 16.15 | 1.4 |
| 8 |  | 2.33 | 0.35 | 6.13 | 28.06 | 1.16 | 0.99 | 19.42 | 2.30 | 10.43 | 0.72 | 6.38 | 17.82 | 0.66 |  |  | 3.25 |  |  |
| 9 |  | 1.21 | 0.43 | 13.51 | 9.51 | 0.67 | 3.93 | 21.10 | 7.60 | 7.08 | 0.51 | 15.27 | 14.46 | 0.63 | 1.32 |  | 2.39 |  |  |
| 10 |  | 1.15 | 0.67 | 9.44 | 1.20 | 0.70 | 6.33 | 33.80 | 7.68 | 7.23 |  | 10.82 | 14.48 | 1.08 |  |  | 5.42 |  |  |
| 11 |  | 4.13 | 0.46 | 4.02 | 28.66 | 0.73 | 2.43 | 20.02 | 1.06 | 8.55 | 3.10 | 3.12 | 17.60 | 0.47 | 0.48 | 1.88 | 3.30 |  |  |
| 12 |  | 5.27 | 0.68 | 4.46 | 29.88 | 1.06 | 1.86 | 20.00 | 1.12 | 8.22 | 2.84 | 3.09 | 16.55 | 0.62 | 0.62 | 1.72 | 2.02 |  |  |
| 13 |  | 1.12 | 0.44 | 10.06 | 1.19 | 0.63 | 6.59 | 40.59 | 7.43 | 3.96 |  | 12.75 | 11.82 | 0.72 |  |  | 2.19 |  |  |
| 16 |  | 1.75 | 1.99 | 4.65 | 18.25 | 3.22 | 0.37 | 8.09 | 3.76 | 17.78 | 1.19 | 2.27 | 10.09 | 1.80 | 3.59 | 9.45 | 11.42 |  |  |
| 17 |  | 0.78 | 2.11 | 3.66 | 19.25 | 1.33 |  | 5.60 | 2.59 | 20.72 | 0.99 | 2.50 | 13.07 | 1.10 | 3.68 | 10.18 | 12.45 |  |  |
| 18 |  | 1.33 | 2.24 | 4.43 | 20.21 | 1.73 |  | 6.52 | 3.17 | 21.70 | 0.80 | 2.31 | 10.59 | 1.12 | 3.27 | 9.22 | 11.36 |  |  |
| 19 |  | 1.56 | 1.99 | 5.03 | 16.92 | 2.86 | 0.30 | 7.67 | 3.56 | 18.42 | 1.12 | 2.59 | 10.02 | 1.75 | 3.91 | 10.11 | 12.18 |  |  |
| 20 |  | 1.15 | 2.33 | 4.53 | 19.44 | 1.52 |  | 5.98 | 2.77 | 22.82 | 0.76 | 2.59 | 10.42 | 1.14 | 3.39 | 9.50 | 11.65 |  |  |
| 21 |  | 1.23 | 1.90 | 4.82 | 21.94 | 2.12 |  | 6.87 | 2.41 | 20.26 | 0.79 | 2.66 | 12.74 | 1.17 | 2.66 | 8.09 | 10.16 |  |  |
| 22 | 0.72 |  | 3.69 |  |  | 2.44 |  |  | 10.66 | 27.47 |  |  |  | 1.75 | 21.11 | 9.31 | 10.86 | 5.78 | 0.76 |
| 24 | 0.51 |  | 2.00 |  |  | 1.68 |  |  | 3.93 | 27.73 |  |  |  | 6.58 | 18.18 | 8.20 | 15.29 | 12.75 | 0.56 |
| 25 |  | 1.13 | 1.88 | 3.18 | 19.51 | 1.10 |  | 9.63 | 1.14 | 14.82 | 1.62 | 2.68 | 15.12 | 1.00 | 4.24 | 10.53 | 12.42 |  |  |
| 26 | 0.95 |  | 4.01 |  |  | 0.41 |  |  | 7.63 | 27.34 |  |  |  | 1.26 | 12.58 | 12.64 | 14.91 | 14.10 | 1.42 |
| 27 |  | 0.93 | 2.66 | 4.68 | 19.20 | 1.14 |  | 3.69 | 3.65 | 25.01 | 0.51 | 2.55 | 8.77 | 0.90 | 3.94 | 10.10 | 12.25 |  |  |
| 28 |  | 1.11 | 1.95 | 5.80 | 15.51 | 1.82 |  | 9.52 | 3.17 | 18.10 | 1.80 | 4.14 | 14.97 | 1.09 | 3.16 | 8.00 | 9.85 |  |  |
| 34 |  | 1.32 | 1.94 | 2.97 | 22.13 | 1.03 |  | 9.12 | 1.37 | 13.17 | 0.57 | 2.09 | 16.42 | 1.00 | 4.29 | 10.49 | 12.07 |  |  |
| 39 |  | 0.94 | 2.11 | 1.77 | 21.20 | 1.59 |  | 7.87 | 2.26 | 18.90 | 0.31 | 1.23 | 15.70 | 0.93 | 3.51 | 9.78 | 11.90 |  |  |
| 42 |  | 2.10 | 2.08 | 4.96 | 19.42 | 2.48 |  | 9.10 | 2.72 | 17.24 | 0.86 | 2.17 | 11.18 | 1.30 | 3.37 | 9.49 | 11.50 |  |  |

i – iso methyl branched, ai – anteiso methyl branched, n – normal i.e. straight chain fatty acids, br – methyl branched, 16:1* monounsaturated C 16 with the undetermined location of the double bond in the molecule, 18:1* monounsaturated C 18 with the undetermined location of the double bond in the molecule.

**Table S3** Results of sequence comparison to NCBI 16S ribosomal RNA database (bacteria and archaea type strains), accessed 2023/02/25. Displayed are the closest database matches based on max score and % sequence identity; in case of several species matching at the same max score, all those individual species are listed

| Strain | Accession | Closest database match species | % sequence similarity to the database entry | Accession |
| --- | --- | --- | --- | --- |
| AKT01 | OQ506581 | *Streptomyces flavovirens*  *S. flavogriseus* | 100 % | NR_112509.1  NR_028988.1 |
| AKT02 | OQ506582 | *Streptomyces microflavus,*  *S. alboviridis* | 99.85 % | NR_043854.1  NR_112340.1 |
| AKT03 | OQ506583 | *Streptomyces sanglieri* | 99.09 % | NR_041417.1 |
| AKT04 | OQ506584 | *Streptomyces flavovirens,*  *S. flavogriseus* | 99.93 % | NR_112509.1  NR_028988.1 |
| AKT05 | OQ506585 | *Streptomyces albiaxialis* | 99.18 % | NR_112599.1 |
| AKT06 | OQ506586 | *Streptomyces microflavus,*  *S. alboviridis* | 99.78 % | NR_043854.1  NR_112340.1 |
| AKT07 | OQ506587 | *Nocardia carnea* | 99.85 % | NR_118200.1 |
| AKT08 | OQ506588 | *Pseudonocardia alni* | 99.56 % | NR_117429.1 |
| AKT09 | OQ506589 | *Streptomyces brevispora* | 99.42 % | NR_117081.1 |
| AKT10 | OQ506590 | *Pseudonocardia alni* | 100 % | NR_117429.1 |
| AKT11 | OQ506591 | *Streptomyces flavovirens,*  *S. flavogriseus* | 99.93 % | NR_112509.1  NR_028988.1 |
| AKT12 | OQ506592 | *Streptomyces flavovirens,*  *S. flavogriseus* | 99.86 % | NR_112509.1  NR_028988.1 |
| AKT13 | OQ506593 | *Pseudonocardia alni* | 99.71 % | NR_117429.1 |
| AKT16 | OQ506594 | *Streptomyces sampsonii,*  *S. hydrogenans*  *S. coelicolor,*  *S. limosus,*  *S. felleus* | 99.93 % | NR_112362.1  AB184868.1  NR_112305.1  NR_112279.1  NR_112266.1 |
| AKT17 | OQ506595 | *Streptomyces violaceolatus* | 100 % | NR_112370.1 |
| AKT18 | OQ506596 | *Streptomyces coelicoflavus* | 99.85 % | NR_041175.1 |
| AKT19 | OQ506597 | *Streptomyces sampsonii,*  *S. hydrogenans,*  *S. coelicolor,*  *S. limosus,*  *S. felleus* | 100 % | NR_112362.1  AB184868.1  NR_112305.1  NR_112279.1  NR_112266.1 |
| AKT20 | OQ506598 | *Streptomyces violaceolatus* | 99.49 % | NR_112370.1 |
|  |  |  |  |  |
| AKT21 | OQ506599 | *Streptomyces coelicoflavus* | 100 % | NR_041175.1 |
| AKT22 | OQ506600 | *Nocardia niigatensis* | 98.04 % | NR_117402.1 |
| AKT24 | OQ506601 | *Nocardia mangyaensis* | 99.20% | KU601231.2 |
| AKT25 | OQ506602 | *Streptomyces griseobrunneus,*  *S. cavourensis,*  *S. bacillaris,*  *Kitasatospora albolonga* | 99.53 % | NR_112577.1  NR_112345.1  NR_041146.1  NR_041144.1 |
| AKT26 | OQ506603 | *Nocardia cavernae* | 98.15 % | NR_157707.1 |
| AKT27 | OQ506604 | *Streptomyces olivaceus,*  *S. pactum* | 99.71 % | NR_112581.1  NR_041134.1 |
| AKT28 | OQ506605 | *Streptomyces songpinggouensis,*  *S. tauricus* | 98.45 % | NR_169378.1  NR_028621.1 |
| AKT34 | OQ506606 | *Streptomyces resistomycificus,*  *S. hydrogenans,*  *S. sampsonii,*  *S. coelicolor,*  *S. limosus,*  *S. felleus,*  *S. griseochromogenes* | 99.78 % | NR_042100.1  AB184868.1  NR_112362.1  NR_112305.1  NR_112279.1  NR_112266.1  NR_042102.1 |
| AKT39 | OQ506607 | *Streptomyces abietis* | 98.55 % | NR_114347.1 |
| AKT42 | OQ506608 | *Streptomyces microflavus,*  *S. alboviridis* | 99.86 % | NR_043854.1  NR_112340.1 |

**Table S4** Identification of actinobacterial strains AKT 2 and AKT 3 in the MALDI Biotyper database depending on incubation time and culture media: blood agar (BL), nutrient agar (NA), tryptic soy-thioglycolate agar (TS), yeast-extract glucose agar (medium79) and brain heart infusion agar BHI). All samples were prepared by the formic acid extraction method. Note: The database used here is the standard MALDI Biotyper database, not the improved *in-house* database for Actinobacteria strains (used for Table 3 results)

| Strain | Culture medium | Incubation time (days) | Organism | Score value |
| --- | --- | --- | --- | --- |
| AKT 2 | BL | 2 | *NR/Bacillus subtilis* | 1.352 |
|  |  | 4 | *NR/Streptomyces avidinii* | 1.42 |
|  |  | 7 | *NR/Streptomyces griseus* | 1.541 |
|  | NA | 2 | *NR/Lactobacillus curvatus* | 1.469 |
|  |  | 4 | *NR/Streptomyces badius* | 1.434 |
|  |  | 7 | *NR/Streptomyces badius* | 1.362 |
|  | TS | 2 | *NR/Streptomyces badius* | 1.585 |
|  |  | 4 | *NR/Streptomyces griseus* | 1.388 |
|  |  | 7 | *Streptomyces badius* | 1.834 |
|  | 79 | 2 | *Streptomyces badius* | 1.783 |
|  |  | 4 | *Streptomyces badius* | 1.758 |
|  |  | 7 | *NR/Streptomyces sp* | 1.204 |
|  | BHI | 2 | *NR/Streptomyces badius* | 1.569 |
|  |  | 4 | *NR/Streptomyces badius* | 1.411 |
|  |  | 7 | *NR/Streptomyces griseus* | 1.459 |
| AKT 3 | BL | 2 | *NR/Streptomyces griseus* | 1.576 |
|  |  | 4 | *NR/Streptomyces badius* | 1.519 |
|  |  | 7 | *Streptomyces badius* | 1.702 |
|  | NA | 2 | *NR/Streptomyces lavendulae* | 1.543 |
|  |  | 7 | *NR/Streptomyces badius* | 1.622 |
|  | TS | 2 | *NR/Streptomyces griseus* | 1.211 |
|  |  | 4 | *Streptomyces badius* | 1.889 |
|  |  | 7 | *Streptomyces badius* | 1.746 |
|  | 79 | 2 | *NR/Streptomyces griseus* | 1.4 |
|  |  | 4 | *Streptomyces badius* | 1.716 |
|  |  | 7 | *Streptomyces badius* | 1.735 |
|  | BHI | 2 | *NR/Streptomyces griseus* | 1.415 |
|  |  | 4 | *NR/Streptomyces badius* | 1.674 |
|  |  | 7 | *Streptomyces badius* | 1.865 |

**Table S5** Comparing MALDI-TOF identification results in Biotyper and an *in-house* database

| AKT no | PCM no | Conditions* | Identification MALDI-TOF Biotyper | Score value | Identification in-house database | Score value | Genus (secure) |
| --- | --- | --- | --- | --- | --- | --- | --- |
| AKT01 | 3204 | 01_NA_48h_e | *Streptomyces griseus* | 1.936 | *Streptomyces flavovirens* | 2.29 | *Streptomyces* |
| AKT02 | 3205 | 02_NA_48h_e | *Streptomyces griseus* | 1.82 | *Streptomyces microflavus* | 2.259 | *Streptomyces* |
| AKT03 | 3206 | 03_NA_48h_e | *Streptomyces badius* | 1.915 | *Streptomyces flavovirens* | 1.776 | *-* |
| AKT04 | 3207 | 04_79_48h_e | *Streptomyces badius* | 1.81 | *Streptomyces flavovirens* | 1.982 | *-* |
| AKT05 | 3208 | 05_BL_72h_e | NR/*Streptomyces violaceoruber* | 1.422 | *Streptomyces microflavus* | 2.185 | *Streptomyces* |
| AKT06 | 3209 | 06_BHI_48h_e  06_NA_72h_DTFA | NR/*Streptomyces griseus*  *Streptomyces badius* | 1.625  1.890 | *Streptomyces microflavus*  *Streptomyces microflavus* | 2.165  2.185 | *Streptomyces* |
| AKT07 | 3210 | 07_BL_96h_e | *Nocardia carnea* | 2.4 | NR/*Nocardia cavernae* | 1.482 | *Nocardia* |
| AKT08 | 3211 | 08_TS_48h_e | Not reliable | 1.46 | *Pseudonocardia alni* | 2.075 | *Pseudonocardia* |
| AKT09 | 3212 | 09_79_48h_e  09_79_72h_DT | NR/*Streptomyces griseus*  *Streptomyces griseus* | 1.536  1.977 | NR/*Streptomyces microflavus*  NR/*Streptomyces microflavus* | 1.179  1.128 | *-* |
| AKT10 | 3213 | 10_BHI_48h_e | Not reliable | 1.454 | *Pseudonocardia alni* | 2.333 | *Pseudonocardia* |
| AKT11 | 3214 | 11_BL_48h_e | NR/*Streptomyces griseus* | 1.482 | *Streptomyces flavovirens* | 1.903 | *-* |
| AKT12 | 3215 | 12_BL_48h_e | Not reliable | 1.538 | *Streptomyces flavovirens* | 1.957 | *-* |
| AKT13 | 3216 | 13_TS_48h_e | Not reliable | 1.532 | *Pseudonocardia alni* | 1.851 | *-* |
| AKT16 | 3217 | 16_NA_72h_e | Not reliable | 1.431 | *Streptomyces sampsonii* | 1.826 | *-* |
| AKT17 | 3218 | 17_79_72h_e | *Streptomyces violaceoruber* | 1.8 | *Streptomyces violaceolatus* | 2.415 | *Streptomyces* |
| AKT18 | 3219 | 18_NA_48h_e | *Streptomyces violaceoruber* | 1.852 | *Streptomyces coelicoflavus* | 2.041 | *Streptomyces* |
| AKT19 | 3220 | 19_NA_72h_e | Not reliable | 1.334 | *Streptomyces sampsonii* | 1.844 | *-* |
| AKT20 | 3221 | 20_79_72h_e | *Streptomyces violaceoruber* | 1.849 | *Streptomyces violaceolatus* | 2.379 | *Streptomyces* |
| AKT21 | 3222 | 21_NA_48h_e | *Streptomyces violaceoruber* | 1.85 | *Streptomyces coelicoflavus* | 2.133 | *Streptomyces* |
| AKT22 | 3223 | 22_TS_48h_e | Not reliable | 1.502 | *Nocardia cavernae* | 1.864 | *-* |
| AKT24 | 3224 | 24_79_48h_e | Not reliable | 1.377 | *Nocardia mangyaensis* | 1.829 | *-* |
| AKT25 | 3225 | 25_NA_48h_e | Not reliable | 1.334 | NR/*Streptomyces microflavus* | 1.047 | *-* |
| AKT26 | 3226 | 26_79_48h_e | Not reliable | 1.305 | *Nocardia cavernae* | 1.815 | *-* |
| AKT27 | 3227 | 27_BL_72h_e | NR/*Streptomyces* sp | 1.339 | *Streptomyces violaceolatus* | 1.773 | *-* |
| AKT28 | 3228 | 28_79_72h_e | Not reliable | 1.302 | NR/*Streptomyces microflavus* | 1.034 | *-* |
| AKT34 | 3229 | 34_79_72h_e | Not reliable | 1.365 | *Streptomyces sampsonii* | 2.125 | *Streptomyces* |
| AKT39 | 3230 | 39_79_72h_e | Not reliable | 1.398 | NR/*Streptomyces flavovirens* | 1.015 | *-* |
| AKT42 | 3231 | 42_NA_72h_e  42_79_48h_DTFA | Not reliable  *Streptomyces griseus* | 1.391  1.961 | *Streptomyces microflavus*  *Streptomyces microflavus* | 1.977  2.187 | *Streptomyces* |

* Conditions: culture media: blood agar (BL), nutrient agar (NA), tryptic soy-thioglycollate agar (TS), yeast-extract glucose agar (medium79) and brain heart infusion agar BHI); e - extraction procedure: ethanol-formic acid (EFAE), DT, direct transfer, DTFA, direct transfer method with formic acid treatment on the target plate.
